# Diffusion tensor imaging point to ongoing functional impairment in HIV-infected children at age 5, undetectable using standard neurodevelopmental assessments

**DOI:** 10.1186/s12981-020-00278-z

**Published:** 2020-05-19

**Authors:** Christelle Ackermann, Savvas Andronikou, Muhammad G. Saleh, Martin Kidd, Mark F. Cotton, Ernesta M. Meintjes, Barbara Laughton

**Affiliations:** 1 Department of Radiodiagnosis, Faculty of Medicine and Health Sciences, University of Stellenbosch, Tygerberg, South Africa; 2 Vice Chair of Research, Department of Paediatric Radiology, Children’s Hospital of Philadelphia, Philadelphia, USA; 3 MRC/UCT Medical Imaging Research Unit, Department of Human Biology, University of Cape Town, Cape Town, South Africa; 4 Centre for Statistical Consultation, University of Stellenbosch, Stellenbosch, South Africa; 5 Family Clinical Research Unit, Department of Paediatrics and Child Health, Faculty of Medicine and Health Sciences, University of Stellenbosch, Tygerberg, South Africa; 6 Tygerberg Hospital, 4th Floor, Cape Town, 7505 South Africa; 7 CHOP, 3401 Civic Centre Blvd., Philadelphia, 19104 USA; 8 Stellenbosch University, Private Bag X1, Matieland, 7602 South Africa; 9 Present Address: Cape Town, 7500 South Africa; 10 UCT Faculty of Health Sciences, Barnard Fuller Building, Anzio road, Observatory, Cape Town, 7935 South Africa; 11 Present Address: Bellville, 7530 South Africa

**Keywords:** HIV, Cognitive functioning, ARV, DTI, Functional anisotropy, White matter

## Abstract

**Background:**

Perinatal HIV infection negatively impacts cognitive functioning of children, main domains affected are working memory, processing speed and executive function. Early ART, even when interrupted, improves neurodevelopmental outcomes. Diffusion tension imaging (DTI) is a sensitive tool assessing white matter damage. We hypothesised that white matter measures in regions showing HIV-related alterations will be associated with lower neurodevelopmental scores in specific domains related to the functionality of the affected tracts.

**Methods:**

DTI was performed on children in a neurodevelopmental sub study from the Children with HIV Early Antiretroviral (CHER) trial. Voxel-based group comparisons to determine regions where fractional anisotropy and mean diffusion differed between HIV+ and uninfected children were done. Locations of clusters showing group differences were identified using the Harvard–Oxford cortical and subcortical and John Hopkins University WM tractography atlases provided in FSL. This is a second review of DTI data in this cohort, which was reported in a previous study. Neurodevelopmental assessments including GMDS and Beery-Buktenica tests were performed and correlated with DTI parameters in abnormal white matter.

**Results:**

38 HIV+ children (14 male, mean age 64.7 months) and 11 controls (4 male, mean age 67.7 months) were imaged. Two clusters with lower fractional anisotropy and 7 clusters with increased mean diffusion were identified in the HIV+ group. The only neurodevelopmental domain with a trend of difference between the HIV+ children and controls (*p *= 0.08), was Personal Social Quotient which correlated to improved myelination of the forceps minor in the control group. As a combined group there was a negative correlation between visual perception and radial diffusion in the right superior longitudinal fasciculus and left inferior longitudinal fasciculus, which may be related to the fact that these tracts, forming part of the visual perception pathway, are at a crucial state of development at age 5.

**Conclusion:**

Even directed neurodevelopmental tests will underestimate the degree of microstructural white matter damage detected by DTI. The visual perception deficit detected in the entire study population should be further examined in a larger study.

## Manuscript

### Background

It is well established that perinatally acquired HIV infection negatively impacts cognitive functioning [1]. The principal domains affected vary with limited data from countries where HIV is prevalent. A recent meta-analysis of 22 studies (37% from sub-Saharan countries) on vertically infected children aged 6 to 18 years, found that the main cognitive domains affected by HIV are working memory, processing speed and executive functioning [2].

Studies show that commencing combination antiretroviral therapy (ART) before 6 months in perinatally HIV-infected (HIV+) infants improves neurodevelopmental and clinical outcomes [3–6]. Concerns that ART might cause neurotoxicity and that adherence may wane, led to planned treatment interruption studies, which showed safety and no effect on short term neurocognition [7, 8]. The clear advantage of early time-limited over deferred-continuous therapy for clinical outcomes was demonstrated in the Children with HIV Early Antiretroviral (CHER) trial [9]. Recently, Laughton et al. reported neurodevelopmental outcomes over 5 years in a CHER sub-study. Neurodevelopmental outcomes were similar between the treatment arms (delayed continuous ART or early ART with interruption at 40 or 96 weeks) and uninfected controls. The only exception was visual perception, measured on the Beery Visual Perception subtest, where all HIV+ arms performed significantly worse. This deficit was neither detectable on the Beery visual motor integration test (Beery-VMI) [10] nor the Griffiths Mental Development Scales (GMDS) [11].

Neuroimaging is instrumental in describing HIV effects on brain macro- and microstructure [12]. Specifically, diffusion tensor imaging (DTI) can be used to examine the nature of white matter (WM) damage through quantitative parameters such as fractional anisotropy (FA) and mean diffusivity (MD) [13–17]. Loss of axonal integrity decreases FA and increases MD, however, increased FA may also indicate diminished complexity of the axonal matrix due to loss of crossing fibres [18]. We previously published a DTI study on HIV+ children (mean age 5.7 years) from the cohort studied by Laughton et al., and demonstrated WM abnormalities of the projectional fibres of the corticospinal tracts (CST) and also association fibres of the superior longitudinal fasciculus (SLF), inferior longitudinal fasciculus (ILF), inferior frontal occipital fasciculus (IFOF) and uncinate fasciculus (UF). Children on continuous ART from within the first year of life had less WM damage than those randomised to treatment interruption. This was an interesting finding when considering that Laughton et al. showed that neurodevelopmental outcome at 5 years was not adversely affected by planned treatment interruption [11, 18].

The aim of the current study was to examine associations of FA and MD values in regions shown to be significantly different between HIV+ children and controls in the aforementioned DTI study [18], with directed neurodevelopmental scores. The latter were identified as those tests related to the expected function of involved WM tracts, for example CST and motor development, as opposed to the full battery of neurodevelopmental tests. We hypothesised that WM measures in regions showing HIV-related alterations would be associated with lower neurodevelopmental scores in specific domains related to the functionality of the affected WM tracts.

## Methods

### Subjects

The study group is uniquely homogeneous in that all the children began ART before 18 months, are from the same socioeconomic background and have a narrow age range.

56 Xhosa children enrolled in a neurodevelopmental sub study of the CHER trial [9, 19] in Cape Town, South Africa underwent magnetic resonance imaging (MRI) of the brain at 5 years of age. The group comprised HIV+ children who commenced ART early and age-matched, HIV-uninfected controls from a parallel vaccine study, with informed consent from parents or caregivers [20].

Inclusion criteria for the neurodevelopmental sub-study were: birth weight > 2000 g, normal neurological examination at a clinical visit near three months of age and no central nervous system problems or dysmorphic syndromes.

### The CHER trial

The CHER trial (the source of our patient population) was a two-centre study in which HIV + infants between 6 and 12 weeks of age with CD4 ≥ 25% were randomized to one of three treatment strategies: ART deferred (ART-Def) until indicated; early limited ART for 40 weeks (ART-40 W); or early limited ART for 96 weeks (ART-96 W). Continuous ART was initiated in ART-Def when the CD4 declined below 25% in the first year of life and 20% thereafter or for Centres for Disease Control severe stage B or C disease. The same criteria applied to restarting ART in ART-40 W and ART-96 W. Infants with a CD4% < 25% were enrolled into a separate group (part B), initially to be randomised into ART-40 W and ART-96 W, but then retained on early continuous ART. The entire cohort comprised 451 HIV + infants below 12 weeks of age, of which 115 were enrolled in Cape Town [9].

First-line ART was lopinavir–ritonavir, lamivudine and zidovudine; only one child was on second line therapy comprising Didanosine, Abacavir with Nevirapine. Most mothers participated in the prevention of mother to child transmission program, which included zidovudine antenatally from 32 weeks and for infants a single dose nevirapine at delivery and zidovudine for 7 days.

### Neurodevelopmental assessments

The GMDS extended revised version (2–8 years) was performed at 5 years of age [21]. The GMDS assesses neurodevelopment on the subscales: locomotor, personal-social, hearing and language, eye and hand co-ordination, performance (visuospatial skills including speed and precision) and practical reasoning (Table 1). A global GMDS score is also obtained. Standardized translations into IsiXhosa and Afrikaans were used. One of two pediatricians conducted the assessments, assisted by a GMDS-trained translator. We converted raw scores into age equivalents using standardized norms and calculated a quotient as a percentage of each child’s chronological age, using the United Kingdom norms with a mean of 100 and standard deviation of 15 [21, 22], Significant developmental delay was regarded as quotients below 70. The Beery-Buktenica tests of visual-motor integration (Beery-VMI), visual perception and motor coordination (6th edition) were also administered (Table 1) [23]. Standard scores were calculated from raw scores using USA norms. While these developmental tests are not standardised for South African children, they are often used and considered culturally fair and reliable [21, 24, 25].

**Table 1:** Description of abilities assessed with the GMDS and Beery-Buktenica test

Griffiths mental development scales	
Subscale	Description of abilities assessed
Locomotor	Balance and stability–jumping over hurdles, balancing on one leg, skipping and running
Personal-social	Self-care including dressing, washing, tying shoe laces and being able to provide full name and address
Hearing and language	Receptive and expressive language is assessed Naming objects and describing their use Children are required to freely talk about a large/busy picture where vocabulary, sentence structure, pronouns and descriptive words are assessed Auditory short-term recall with repetition Naming colors, similarities opposites and descriptive
Eye and hand Co-ordination	Free drawing of a person and a house. Copying geometric shapes Writing name and copying letters Cutting and folding paper and threading beads
Performance	Visuo-spatial skills including speed and precision Completing form boards and block patterns which are timed
Practical reasoning	Closest to arithmetical reasoning: counting blocks, knowing days of the week, high/low, long/short, heavy/light, middle and concept of speed. Short term memory of items shown Arranging sequences of cards to tell a story
General Griffiths Quotient:	Average of the 6 subtests above

Beery-Buktenica test of visual motor integration	
Beery VMI	Child is required to copy various geometric forms and draw them below the example figure
Beery motor Co-ordination	Draw the same geometric forms by joining dots and keeping within the guidelines. Draw as many as can within a time limit
Beery visual perception	Identify shape out of a few that matches the example. Do as many as can within a time limit

Baseline laboratory and clinical data at enrolment and within 6 months of MRI scan, including CD4, CD8 parameters and viral load (VL) were obtained from the CHER database. VL > 750,000 copies/mL were assigned as 750 001 and those < 400 copies/mL as 399 (viral suppression).

### Ethics approval for the study was obtained from ethics boards of all institutions involved

#### MRI data acquisition

The children were imaged on a 3T Siemens Allegra MRI (Erlangen, Germany), without sedation while watching an age-appropriate feature film, using structural T1 imaging followed by 2 DTI acquisitions with opposite phase encoding directions using a twice-refocused spin echo sequence [26]. The 3D echo planar imaging (EPI) navigated [27] multiecho MPRAGE [28] (MEMPR) sequence was acquired in a sagittal orientation with the following parameters: FOV 224 × 224 mm, 144 slices, TR 2530 ms, TE 1.53/3.19/4.86/6.53 ms, TI 1160 ms, flip angle 7°, voxel size 1.3 × 1.0 × 1.0 mm^3^. DTI was performed in 30 directions with b-value 1000 s/mm^2^, voxel size 2 × 2×2 mm^3^, TR/TE 9500/86 ms, and 4 volumes with b = 0 s/mm^2^.

MRIs of children with motion corruption, showing incidental brain abnormalities, interslice instabilities or with an interval of over a year from the GMDS were excluded.

### Data analysis

DTI data were previously analysed and reported in a study describing the distribution and nature of white matter abnormalities as well as the benefit of early treatment [18].

#### Preprocessing

Diffusion weighted volumes with signal dropout or motion corrupted slices were removed, and diffusion encoding scheme adjusted, with a constraint that the same volumes be removed in both DTI acquisitions. Co-registration and susceptibility correction were performed. Briefly, co-registration of individual volumes to the first unweighted image was performed using linear affine (12 degrees of freedom) transformation (FLIRT) in FSL (Oxford Centre for Functional Magnetic Resonance Imaging of the Brain, Oxford, UK). Subsequently, these images were imported to MATLAB (Mathworks, Natick, MA) for susceptibility correction and outlier rejection. Outliers of each acquisition were examined by first calculating z-scores based on 25 and 75 percentile limits; data points above 3 standard deviations beyond the mean were discarded. The two acquisitions were combined into a single corrected image; FA, MD and eigenvalue (e_1_, e_2_, and e_3_) images were generated. The first eigenvalue (e_1_) was AD; the remaining two were used to compute RD (e_23_ = [e_2_ + e_3_]/2).

#### Coregistration

The FA images were first co-registered to corresponding structural images to achieve intra-subject alignment. Structural images of all subjects were then co-registered to a ‘most representative’ control image, then subsequently co-registered to the National Institutes of Health paediatric MRI Data repository T1-template image for children aged 4.5–8.5 years with isotropic resolution 1.0 × 1.0 × 1.0 mm^3^ using linear (FLIRT) and non-linear (FNIRT) co-registration algorithms in FSL. FA images were warped using the same transforms for inter-subject alignment. The same transforms were applied to MD, AD and RD images. A WM binary mask was generated for each subject by applying a FA threshold of 0.2. Individual masks were multiplied to generate a final binary image representing WM regions where FA ≥ 0.2 in all subjects. The binary image was multiplied with the co-registered FA and MD images of each subject to localise statistical analyses, explained below, to the same WM regions.

Voxel-based group comparisons were performed in FSL to determine regions where FA and MD differed significantly between HIV + and control children. To account for multiple comparisons when determining significant clusters, AFNI’s AlphaSim command was used with overall significance level α = 0.05 and individual voxel-wise significance level *p* = 0.01. FWHM values ranged between 3.8 and 5.2 mm across the masked thresholded WM masks and we performed 5000 Monte Carlo simulations. Clusters of at least 258 mm^3^ were significant at these levels.

Locations of clusters showing group differences were identified using the Harvard–Oxford cortical and subcortical and John Hopkins University WM tractography atlases provided in FSL and an MRI atlas of human WM anatomy [29, 30], For each cluster, average FA and MD, and corresponding AD and RD values, were extracted.

### Statistical analysis

Categorical variables were summarised using frequency and percentage frequency distributions overall and by group. Continuous measurements were summarised using means and standard deviation. Variables were compared between the groups using Chi square tests and ANOVA.

Specific functionality of the WM tracts with clusters of abnormal FA and MD in the HIV+ group compared to controls, were identified, right and left sided clusters were analysed separately. We used a directed approach to select neurodevelopmental tests that would closely match this functionality for correlation.

Spearman correlation was used to test for relationships between directed developmental scores and FA and MD values in affected regions. Correlations were performed as a combined group (HIV+ and controls) as well as controls and HIV + groups separately.

## Results

Seven of 56 children assessed were excluded: one HIV + child whose structural image was motion corrupted, one control child with incidental periventricular leukoencephalopathy, two HIV+ children with data interslice instabilities, 2 control children in whom GMDS at age 5 were not performed, 1 HIV + child with a period of more than 1 year and 2 months between the Griffiths analysis and the MRI scan. We therefore present data for 38 HIV + children (mean ± SD = 5.4 ± 0.3 years; 14 boys) and 11 healthy controls (mean ± SD = 5.6 ± 0.5 years; 4 boys, 9 HIV exposed) (Table 2).

**Table 2:** Demographics and neurodevelopmental scores of the HIV+ group and controls

	HIV+	Controls	*p*
N	38	11 *	–
Gender (M/F)	14/24 (37%/63%)	4/7 (36%/64%)	0.97 (χ^2^)
Age at Griffiths (months)	60.9 (1.3)	63.3 (4.8)	0.12
Range	58.0–64.5	59.5–71.0	–
Age at scan (months)	64.7 (3.5)	67.7 (5.5)	0.11
Range	58.8–74.4	61.2–74.4	–
Time between Griffiths and scan (days)	119.2 (104.3)	128.3 (131.8)	0.81
Range	0–386	21–392	–
Age starting ART (weeks)	18 (16.8)	NA	–
Range	7.0–75.7		–
Cumulative Time on ART (weeks)	234 (50.8)	NA	–
CD4		NA	–
Baseline	1965.4 (939.8)	–	–
Nadir	703.2 (391.6)	–	–
At scan	1132.7 (480.7)	–	–
CD4%		NA	–
Baseline	34.5 (10)	–	–
Nadir	20.4 (6.5)	–	–
At scan	35.2 (8.2)	–	–
CD8		NA	–
Baseline	1693.3 (899.3)	–	–
Nadir	575.4 (329.1)	–	–
At scan	1025.7 (536.9)	–	–
Griffiths Q scores			
Locomotor	96.1 (16.2)	93.0 (11.9)	0.57
Personal social	90.8 (9.4)	96.4 (9.0)	0.08
Language	75.4 (10.7)	77.7 (10.7)	0.53
Eye hand coordination	85.4 (9.0)	84.3 (12.2)	0.74
Performance	75.4 (10.5)	78.0 (19.5)	0.57
Practical reasoning	76.7 (8.6)	75.2 (11.4)	0.65
General	83.5 (6.5)	84.1 (8.3)	0.81
Beery-Buktenica			
Visual motor integration	91 (9.1)	87.4 (7.2)	0.23
Visual perception	76.7 (14.7)	83.1 (13.7)	0.22
Motor co-ordination	94.8 (8.3)	93.2 (10.4)	0.59

Values: mean (standard deviation) unless otherwise stated

* 9 HIV exposed and uninfected, 2 unexposed

The HIV + and control groups did not differ for demographic variables (all *p*’s > 0.1); or on the interval between scan and Griffiths, which was 123.8 days on average. The groups did not differ on the GMDS (all *p*’s > 0.5), or the Beery Buktenica motor co-ordination (*p* = 0.59) except for Personal Social Quotients that tended to be lower in HIV+ children (*p *= 0.08). The Beery Buktenica Scales for visual motor integration (*p* = 0.23) and visual perception (*p* = 0.22) also tended to be lower in the HIV + group. The fact that these results were not significant at 5% could be due to a power problem (56% for personal social and 37% for Beery Buktenica scales using independent samples *t* test) but it did show the expected trends.

### Correlation of FA and MD with WM-directed neurodevelopmental tests

**Fig. 1:**
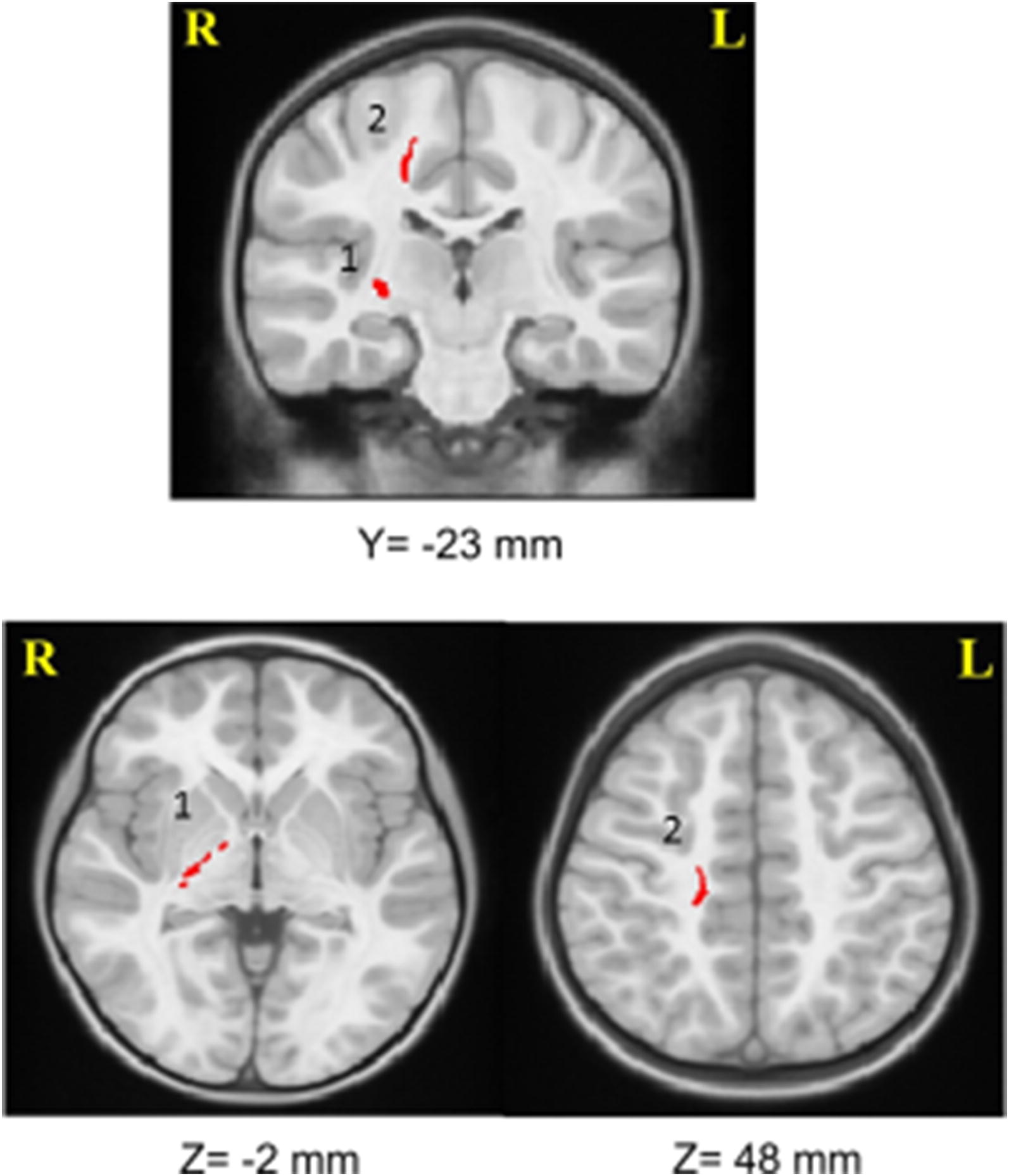
Two clusters in the right corticospinal tract, where FA was lower in HIV+ children than in controls. (1 = right internal capsule, 2 = right parietal lobe)

**Fig. 2:**
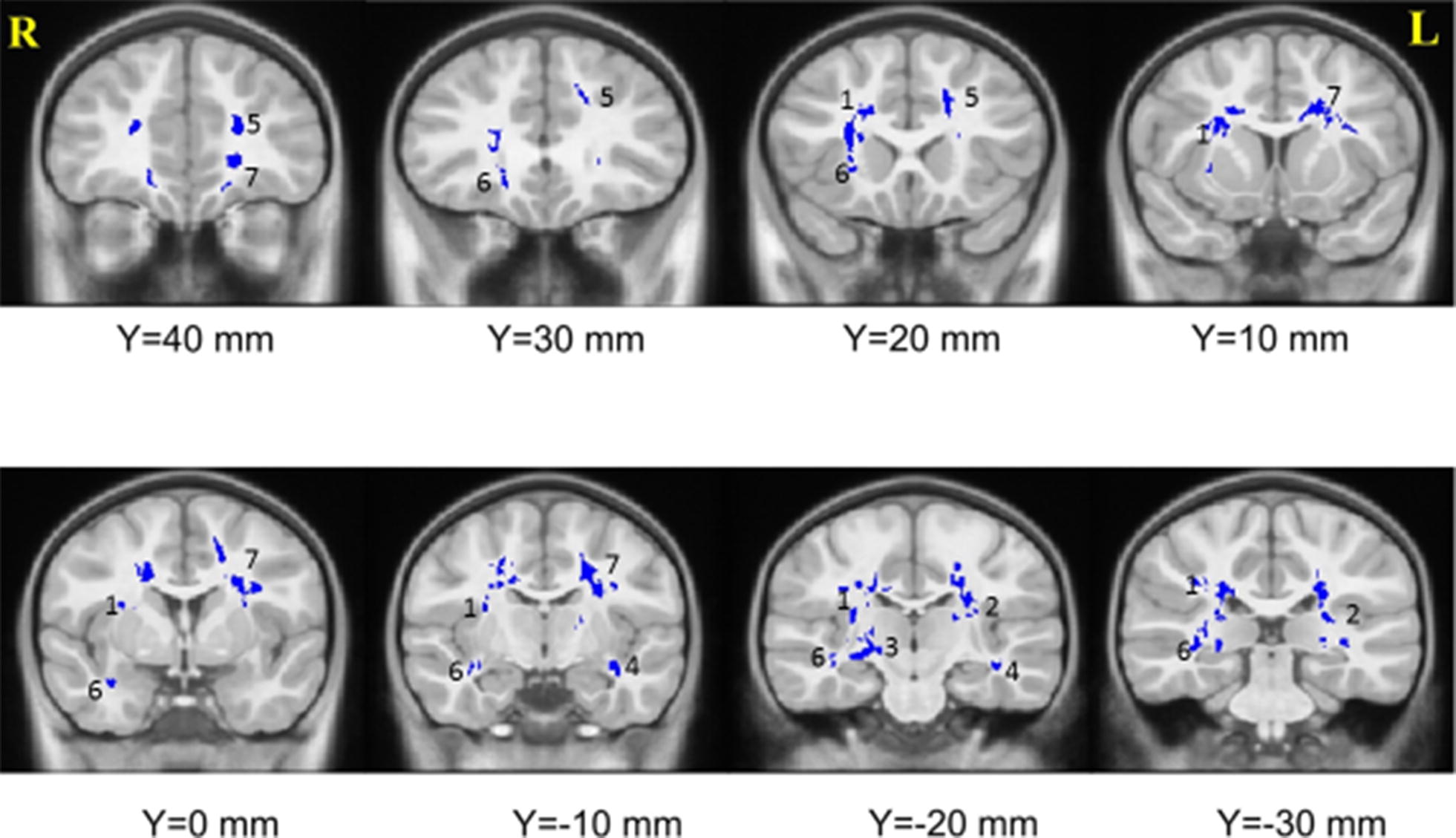
Seven clusters with higher MD in infected children compared to controls. (1 = right SLF, 2 = left ILF, 3 = right CST, 4 = left IFOF, 5 = left forceps minor, 6 = right uncinate fasciculus, 7 = left forceps minor)

Previously, we found lower FA in CST, and higher MD in ILF, SLF, CST, IFOF, forceps minor and UF, in HIV+ children than controls (Figs 1 and 2) [18].

**Fig. 3:**
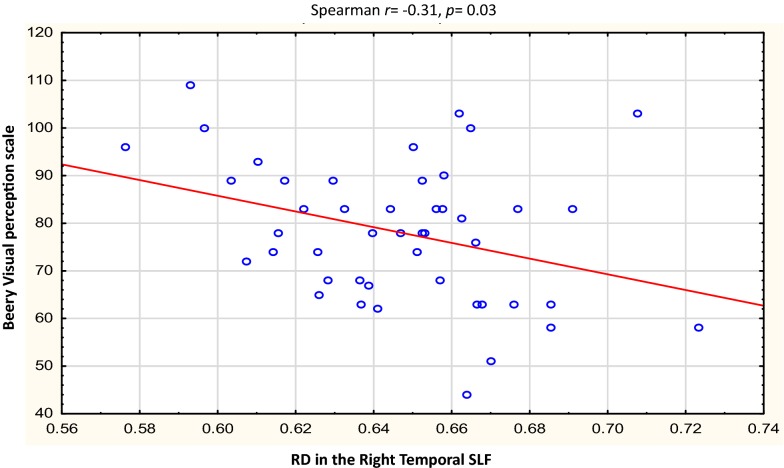
Correlations of FA and MD with WM-directed neurodevelopmental tests.  Overall, increasing RD in the right temporal SLF was associated with poorer performance on Beery visual perception

**Fig. 4:**
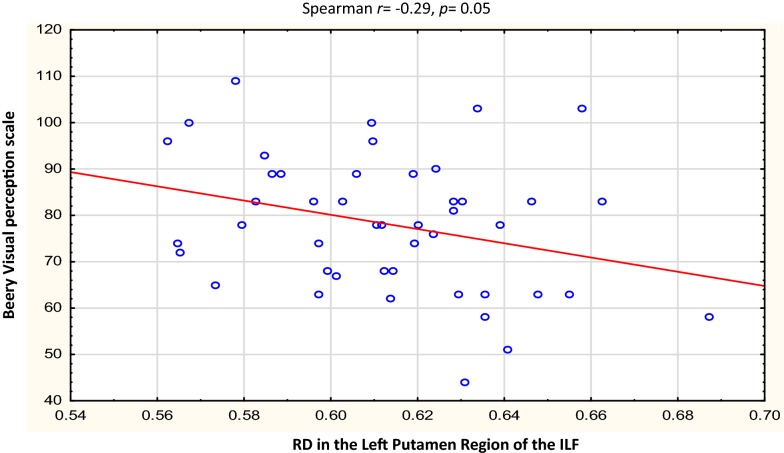
Correlations of FA and MD with WM-directed neurodevelopmental tests.  Overall, increasing RD in the left putamen region of the ILF was associated with poorer performance on Beery visual perception

**Fig. 5:**
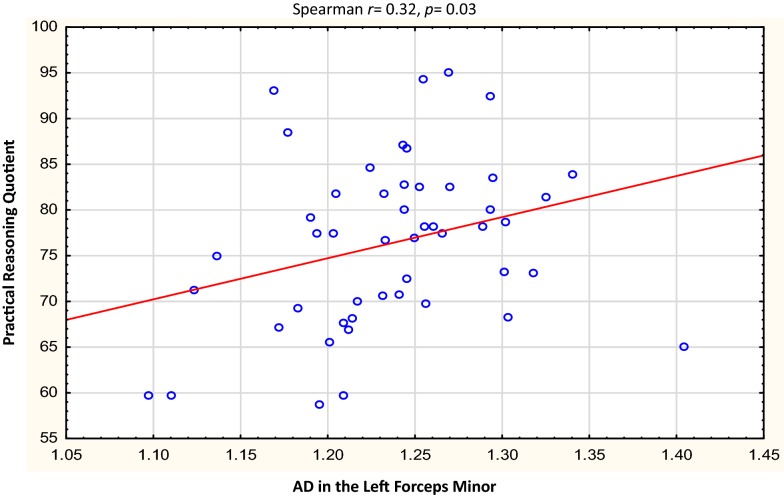
Correlations of FA and MD with WM-directed neurodevelopmental tests. Overall, higher AD in the left forceps minor was related to better practical reasoning

**Fig. 6:**
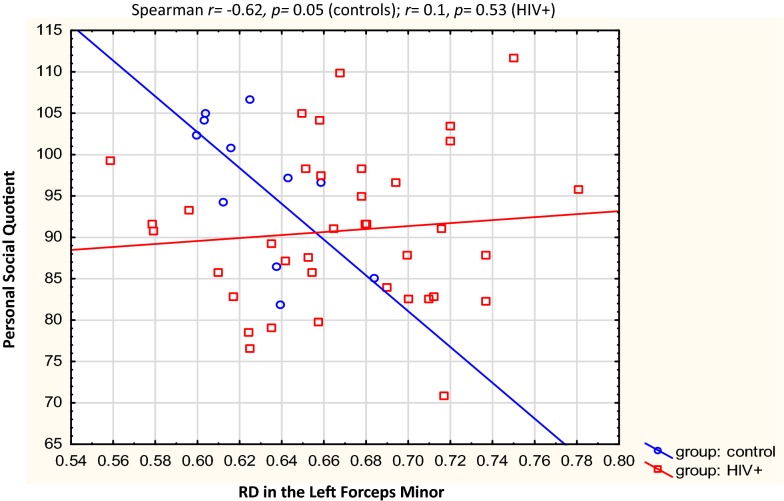
Correlations of FA and MD with WM-directed neurodevelopmental tests. In the controls only, decreased RD in the left forceps minor was strongly related to better personal-social performance

**Fig. 7:**
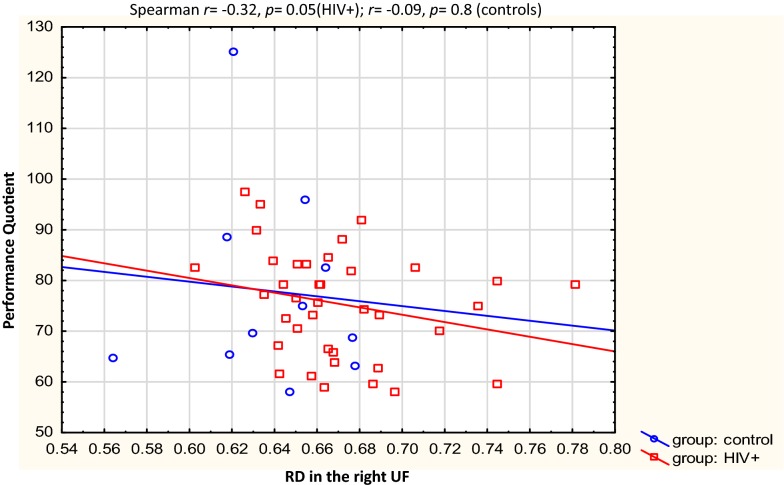
Correlations of FA and MD with WM-directed neurodevelopmental tests. In the HIV+ children only, a negative correlation was found between performance and RD in the right uncinated fasciculus

**Fig. 8:**
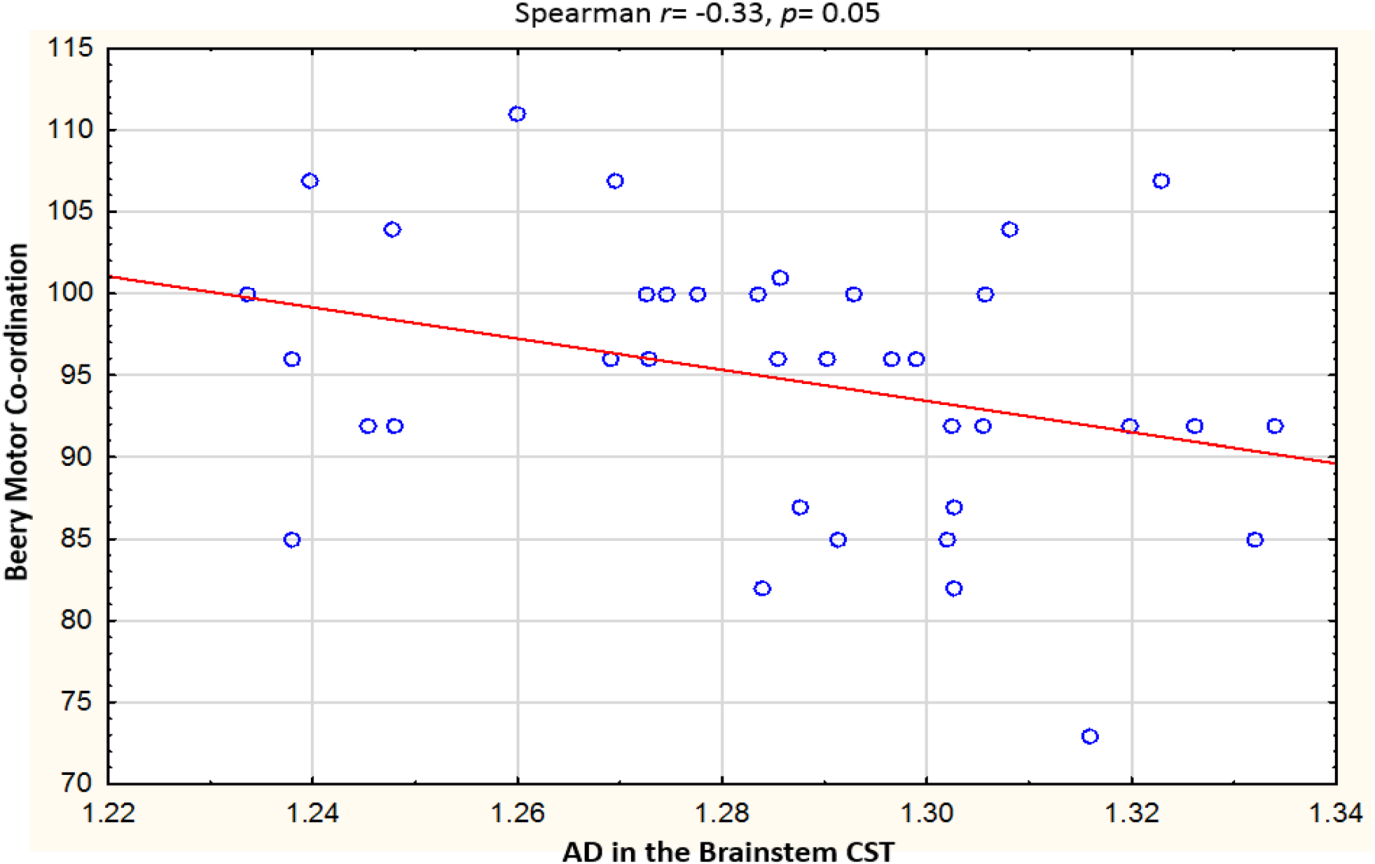
Correlations of FA and MD with WM-directed neurodevelopmental tests. In HIV+ children only, a negative correlation was found between Beery Motor Coordination and AD in the brainstem in the CST

Table 3 describes the clusters and neurodevelopmental tests selected for correlation. Overall, the Beery visual perception was negatively correlated with RD in the right temporal SLF (*r* = − 0.31, *p* = 0.03) and left putamen region of the ILF (*r* = − 0.29, *p *= 0.05). In left forceps minor, higher AD was related to increases in Practical Reasoning scores (*r *= 0.32, *p* = 0.03,), while RD in the same region showed a strong negative correlation with Personal-Social scores in the controls *only* (*r* = − 0.62, *p* = 0.05). In the HIV + group, negative correlations were found between Performance subscale scores and RD in the right UF (*r* = − 0.32, *p* = 0.05), and between Beery Motor Coordination and AD in the brainstem in the CST (*r *= − 0.33, *p* = 0.05). See Figs. 3, 4, 5, 6, 7, 8.

**Table 3:** WM tracts in which clusters showing FA reductions and MD increases in HIV + children compared to controls are located, the function of the implicated tracts and neurodevelopmental tests that assess said function

Tract	Tract function [53]	Griffiths mental development scales Subscale used	Beery-Buktenica test of visual motor integration
Corticospinal Tract	Descending projection fibres connecting motor area to the spinal cord. Arise from motor cortex of pre- and postcentral gyrus	Locomotor	Beery motor Co-ordination
Superior Longitudinal Fasciculus	Association fibres—unite different cortical areas within the same hemisphere. Bidirectional bundles connecting the frontal lobe to the parietal, temporal and occipital lobes Function: integration of auditory and speech nuclei, spatial awareness and symmetric processing Interruption decreases the ability to repeat spoken language and can also cause unilateral neglect	Hearing and Language Eye and Hand Coordination Performance	Beery—VMI Beery—Visual Perception
Inferior Longitudinal Fasciculus	Connects the cortices of the anterior temporal and posterior occipital lobe and joins the inferior aspect of the SLF Function: visual emotion and visual memory Interruption may result in unilateral visual neglect, visual amnesia and hallucinations and also visual hypo emotionality	Eye and Hand Coordination Performance Practical Reasoning	Beery—VMI Beery-visual perception
Inferior Fronto Occipital Fasciculus	Connects the ipsilateral frontal and occipital, posterior parietal and temporal lobes. Function: integration of auditory and visual association cortices with the prefrontal cortex	Personal-Social Language Practical Reasoning	Beery—VMI Beery-Visual Perception
Forceps Minor	The forceps minor is the anterior part of the corpus callosum, it connects the homologous regions of the anterior frontal lobes between two hemispheres. Among the regions included are the front polar cortex which has been shown to be important for cognitive behavioural control, decision making, and attention control	Personal-Social Language Performance Practical Reasoning	
Uncinate Fasciculus	Connects the orbital and inferior frontal gyri rectus to the anterior temporal lobe. It has the longest period of development in terms of FA and is the only WM tract that continues to develop beyond 30 years Part of the limbic system Integrity of the tract has been related to proficiency in auditory-verbal memory and declarative memory	Language Performance Practical reasoning	

*R* right, *L* left, *CST* corticospinal tract, *SLF* superior longitudinal fasciculus, *ILF* inferior longitudinal fasciculus, *IFOF* inferior fronto occipital fasciculus, *UF* uncinate fasciculus

## Discussion

In this study we aimed specifically to examine the potential role of HIV-related WM alterations on neurodevelopmental outcomes, by examining correlations of WM measures with scores on functional domains where affected tracts play a critical role. In view of the increases in RD and AD observed in HIV+ children the associations point to impairment in visual perception, motor coordination and performance (which essentially test visuospatial skills), all of which have been described in HIV+ children [2, 31].

In contrast to most previous studies that demonstrated clear differences between HIV+ and uninfected children on various functional domains [12, 32, 33], children in our study performed similarly at this age on all administered tests. Even in the larger group from the CHER neurodevelopmental sub-study, only visual perception deficits were detected in the HIV+ children at age 5 years [11]. Our findings support those of a recent meta-analysis which concluded that both general intellectual functioning and motor coordination are less impaired in HIV+ children than previously believed [2]. Various factors may explain the different study outcomes, including differences in methodologies, study populations and treatment regimens. The sample studied here comprised children of similar age and socio-economic background with a very well documented treatment history. All had commenced treatment by 18 months and achieved viral suppression by 49 months (median age at first viral suppression was 10 months). In view of the homogeneity and early treatment of these children, it is perhaps not surprising that their neurodevelopmental scores were minimally impaired compared to controls. Alternatively, it is possible that the battery of tests selected are not sensitive enough to detect the subtle impairments or that the sample size was too small to determine differences evident at this age.

It is known that WM integrity is correlated with cognitive performance in a fibre specific manner. [34]. For example, in stroke patients, the degree of CST injury, defined by DTI, correlates with motor impairment [35–38]. The SLF plays an important role in higher brain functions particularly language, [39, 40], spatial awareness and symmetric processing [41]. The ILF is involved in visual memory [42, 43] and the UF in the formation and retrieval of memories [34, 44]. Studies in patients with multiple sclerosis also found a relationship between working memory performance and fibres of the SLF and IFOF [45]. The negative correlation found here between visual perception and RD in the SLF and ILF, provides additional evidence that these tracts have a role in interpreting visual information. Although in our small sub-sample, we did not detect group differences on Beery Visual Perception test, this domain did show HIV-related deficits in the larger sample assessed in the CHER neurodevelopmental sub-study [11]. Notably, the control group in our sub-sample comprised largely HIV-exposed uninfected children (HEU), which may also be affected by perinatal HIV and ART exposure and explain our failure to detect developmental differences on this domain.

Visual perception, encompassing the appreciation of an object’s qualities and its location in space, is dependent on the processing of visual information in the inferior temporal and posterior parietal cortices, respectively [46]. If perception is incorrect or altered in any way, problems with reading, spelling, handwriting, mathematics and comprehension can occur.

Ventral (occipitotemporal) and dorsal (occipitoparietal) visual pathways exist which are functionally specialized. Dorsal stream functions are related to spatial processing and control of visually guided actions and ventral stream functions to perceptual identification [46, 47] The most important WM trajectories of the ventral stream are the ILF and the IFOF [47] also described as intrahemispheric visual association WM tracts, as well as the UF [48]. It is striking that all three of these tracts demonstrated abnormalities in our HIV+ children.

The ventral visual stream is almost adult-like at 5–7 years of age, with DTI metrics demonstrating a rapid increase in FA and decrease in MD in the ILF between ages 5–7 years [47], placing the children in our study at a critical age in maturation of WM tracts for visual perception. HIV-associated WM damage described as being predominantly altered myelination [18], may well account for the abnormalities in visual perception identified by Laughton et al. and the trend demonstrated in the Beery Visual perception test in this group.

Increased AD in the left forceps minor was associated with improved practical reasoning for the group as a whole. Paradoxically, this could lead one to assume that HIV+ children may have better practical reasoning skills. Notably, we failed to detect a group difference for this domain in the current sample or the larger CHER substudy. The Practical Reasoning subscale assesses earliest arithmetic comprehension and the ability to solve very basic practical problems. The forceps minor connects the lateral and medial surfaces of the frontal lobes and crosses the midline via the genu of the corpus callosum. It is an interhemispheric sensory and auditory connection pathway involved in emotional functions and behavioural control [49, 50]. It may play an important role in mathematical skills as indicated in a study where children with increased mathematical ability demonstrated higher FA in WM tracts, particularly the forceps minor and major tracts connecting the frontal lobes with basal ganglia and parietal regions [51]. In our study however, we did not find higher FA but rather increased MD in the forceps minor.

When comparing neurodevelopmental performance between HIV+ and uninfected groups, only the Personal-Social Quotient showed a trend of being lower in HIV+ children (*p *= 0.08). However, this finding does not appear to be attributable to observed WM deficits, as WM measures from clusters in neither the IFOF nor the forceps minor showed association with Personal-Social scores. The strong negative correlation between RD in left forceps minor and Personal Social Quotient among controls suggest that increased myelination (characterised by RD reductions) in this tract may relate to improved personal and social development.

We acknowledge that the sample size (particularly of the control group) is small which may influence the power and increase the possibility of type II errors. The personal-social subscale is the most subjective test and relies on caregiver report for many self-care items. P-values for comparison of the neurodevelopmental scores on other subscales between the HIV+ and control children are all above 0.5 even as high as 0.81 for the General quotient.

Among HIV+ children, increased RD in the right frontal UF is associated with lower scores on the Performance subscale which assesses visuospatial skills, speed and precision. The UF, which connects the hippocampus and amygdala in the temporal lobe with the orbitofrontal cortex, is involved in working memory. This domain was identified as being affected by HIV in the meta-analysis by Phillips and colleagues [2]. It could be that this component of visuospatial processing leads to impaired performance only in the children with the highest RD’s, who were from the HIV+ group. Unfortunately working memory was not assessed as a separate domain in our study.

Increased AD in the brainstem region of the CST is associated with poorer Beery motor coordination test scores in the HIV+ children. Three clusters with abnormal FA and MD in the CST compared to controls were found in the HIV+ group, however no performance differences were found in either the GMDS motor function or the Beery motor coordination. Locomotor deficits were present in this group at a younger age [11] therefore these findings may suggest that conventional neurodevelopmental assessments at this age are not sensitive enough to detect persistent deficits.

Notably, the WM deficits in the UF and brainstem region of the CST (regions that demonstrate association with performance measures in the HIV + group only) were not evident when the children in the current study were re-assessed at 7 years [52], indicating that these deficits may represent a developmental delay that resolves at later ages. In contrast, WM alterations in the ILF and forceps minor persist at age 7 years, suggesting that effects on visual perception may be more long-term or even permanent.

Limitations of this study are the small sample size of controls and the large number of HEU children (9 out of 11) in this group. The secondary effects of HIV and ART exposure in the HEU controls may have influenced neurodevelopmental scores and decreased our ability to detect neurodevelopmental differences between the infected and uninfected groups. On the other hand, this allowed for control of prenatal PMTCT drug administration and other factors such as living in a home affected by HIV. The study group is uniquely homogenous for age, timing of ART and socioeconomic background and the cultural similarity also decreases bias between the groups.

## Conclusion

Although the detrimental effect of HIV on WM is ameliorated by early ART, regional WM alterations on DTI MRI remain and show association at age 5 years with specific functional domains, including visual perception, performance, and motor coordination. In view of the visual perception deficit reported in these children at this age, the effect of HIV on the visual perception pathway should be further examined in a larger study group. Our findings suggest that brain imaging is more sensitive for subtle alterations from HIV and/or ART than standard neurodevelopmental tests.

## Supplementary information

**Additional file 1.** The dataset supporting the conclusions of this article is included within the article as an additional file nr.1. (.xls 5 year old tabular data).

## Data Availability

All data generated or analysed during this study are included in this published article as Additional file 1.

## Abbreviation

ART: Combination antiretroviral therapy
CHER: Children with HIV early antiretroviral trial
Beery-VMI: Beery visual motor integration test
GMDS: Griffith’s mental developmental scales
DTI: Diffusion tensor imaging
WM: White matter
FA: Fractional anisotropy
MD: Mean diffusivity
RD: Radial diffusion
AD: Axial diffusion
CST: Corticospinal tract
SLF: Superior longitudinal fasciculus
ILF: Inferior longitudinal fasciculus
IFOF: Inferior frontal occipital fasciculus
UF: Uncinate fasciculus
MRI: Magnetic resonance imaging
ART-Def: ART deferred
ART-40W: Early limited ART for 40 weeks
ART-96W: Early limited ART for 96 weeks
VL: Viral load
ANOVA: Analysis of variance
HEU: HIV exposed uninfected
